# Accidental exposures to blood and body fluids among health care workers in a Referral Hospital of Cameroon

**DOI:** 10.1186/s13104-016-1923-8

**Published:** 2016-02-15

**Authors:** Julienne Stéphanie Nouetchognou, Jérôme Ateudjieu, Bonaventure Jemea, Dora Mbanya

**Affiliations:** Denis & Lenora Foretia foundation, P.O. Box 14315, Yaoundé, Cameroon; Better Access to Health Care (BAHCare)/Meilleur Accès aux Soins de Santé (M.A. SANTE), P.O. Box 33490, Yaoundé, Cameroon; Department of Biomedical Sciences, University of Dschang, P.O. Box 67, Dschang, Cameroon; Division of Operations Research, Ministry of Public Health, Yaoundé, Cameroon; Yaoundé University Teaching Hospital, Yaoundé, Cameroon; Faculty of Medicine and Biomedical Sciences, University of Yaoundé I, Yaoundé, Cameroon

**Keywords:** Accidental exposure, Health workers, Blood and body fluids, Cameroon, Yaoundé University Teaching Hospital

## Abstract

**Background:**

Accidental exposure to blood and body fluids is a public health concern, especially among health workers and constitutes a risk of transmission of blood-borne viruses including HIV, hepatitis B virus and hepatitis C virus. The objective of this study was to determine the frequency and the post exposure management of accidental exposures to blood and body fluid among health workers in the Yaoundé University Teaching Hospital.

**Methods:**

It was a cross-sectional hospital-based study conducted from the 1st to the 30th of September 2013. Self-administered questionnaires to health workers were used to collect data on self-reported accidents, circumstances and post-exposure management. Their knowledge on accidental exposure to blood was also assessed. Data were entered and analyzed using Epi Info software version 3.5.4. Descriptive analysis was performed to measure the importance of AEB and to evaluate the risk of contamination.

**Results:**

One hundred and fifty health workers were interviewed among which 36.7 % reported having been exposed to blood and body fluid at least once in the preceding 3 months. Splash was the most reported injury (in 60.3 % of cases), followed by needle stick (28.7 %) and cuts (10.9 %). Moreover, 43.6 % of victims were not vaccinated against HBV, 7.3 % were not wearing gloves during the accident and 41 % of splash occurs on injured skin. The majority of victims belong to the surgical Department [20 %, p = 0.2310]. None of these injuries had been reported in the registry of accidental exposure to blood.

**Conclusions:**

There is a high rate of accidental exposure to blood and body fluid in the daily hospital routine. Preventives measures, including wearing of protective equipment’s during care and vaccination against HBV are not systematically done among health workers. Health institution should develop and provide standard operating procedures targeting surveillance of occupational risks, staff training, and supervision.

## Background

Accidental exposure to blood (AEB) is the unintended contact with blood and or body fluids mixed with blood, during a medical intervention. It carries the risk of infection by numerous blood-borne viruses [1, 2]. Health care workers (HCWs) are in direct risk of being infected with disease transmitted by blood during their work due to exposure to biological material and patient’s body fluids during needle injuries or injuries following, cutting, biting or splashing incidents [3]. Although there are numerous blood-borne viruses, hepatitis B virus (HBV) and hepatitis C virus (HCV), as well as human immunodeficiency virus (HIV) are pathogens of greatest concern for HCWs [4]. It is estimated that the risk of HIV infection after needle stick injury is approximately 0.3 %, of hepatitis B infection 30 %, and of hepatitis C 3 % [5, 6]. The frequency of needle stick injuries and the increase prevalence of these blood-borne diseases in general population have a significant impact on the infection risk among HCWs [7].

The World Health Organization (WHO) estimates that 3 million percutaneous exposures occur annually among 35 million HCWs globally; over 90 % occurring in resource constrained countries [8]. Health-care workers in Africa suffer two to four needle-stick injuries per year on average [9]. A study conducted in West Africa estimated the incidence of AEB at 1.8/surgeon/year, 0.6/nurse/year and 0.3/physician/year [10]. In Cameroon, a study conducted in 1997 shows that 60.7 % of HCWs experienced at least one episode of AEB for a one year period [11].

Worldwide occupational exposure accounts for 2.5 % of HIV cases and 40 % of Hepatitis B and C cases among HCWs [12]. Each year as a consequence of occupational exposure, an estimated 66,000 Hepatitis B, 20 million hepatitis C and up-to 260,000 HIV infections occur [13]. On the other hand, the assessment and treatment of the consequences of such accidents is a huge burden on society in terms of the costs of treatment and the absence from work, as well as of the distress and anxiety at work [14–17]. These infections are preventable through infection control measures which significantly reduce the risk of HIV and Hepatitis transmission among health workers [18]. However, studies conducted with the aim to evaluate the reporting of accidents have shown that the compliance with the standard precautions amongst HCWs are low as well as that the propensity to avoid medical assistance after accidents is very frequent [19].

In the control of hazards facing health workers, baseline and periodic assessment of exposure to these hazards is an important strategy which is useful as a decision making tool in risk assessment and management of occupational hazards. Most developing countries, Cameroon included, may not have surveillance system for occupational exposure to blood and body fluids, hence limiting estimation of the exact magnitude of the problem, description of the management of the case and a risk assessment of the AEB. The frequency of the occurrence of AEB was determined, knowledge assessed, actions undertaken after the exposures described, and risk of contamination among healthcare workers in University teaching hospital of Yaoundé, Cameroon assessed.

## Methods

### Study design

It was a cross-sectional hospital-based study conducted in the Yaoundé University Teaching Hospital (YUTH) from the 1st to the 30th of September 2013. It was an exhaustive sampling of all HCWs assigned in services that receive and take care of patients.

### Study population

The study population consisted of health-care workers who came into contact with patients, or were potentially exposed to body fluids from patients during their jobs. These healthcare workers included physicians (resident doctors and intern doctors), nurses and nurse assistant, laboratory personnel. Those who were present at the time of data collection were recruited. Staffs from administrative services, pharmacists, security guards, drivers and all who refused to participate in the study were not included.

### Study setting

The YUTH is a hospital of the third level of the health pyramid, with a patient load of 500–1000 seen each week and it has a relatively high number of staff, compared to other institutions of the city. At the time of the study they were 325 nursing staff consisting of 62 physicians, 236 nursing staff (including 98 nurses, 63 nurse assistants and 75 nursing aids), and 27 laboratory technicians.

### Study procedure

A questionnaire with closed and open-ended questions was prepared for the purpose of this study. It had 30 questions, including those about demographic characteristics (age, gender, occupation, job, length of employment), specific questions on the types and numbers AEB encountered within the last 3 months, as well as measures taken after the accident. In addition, the questionnaire was designed to obtain information on vaccination status for hepatitis B and knowledge of diseases that may occur following to an AEB. The questionnaires were pre-tested for validity, meaning, comprehension of questions in 20 sampled health workers of the Biyem-Assi District Hospital in Yaoundé, and appropriate modifications made. The questionnaire was anonymous and self-administrated. A convenience sampling method was adopted in each service, receiving all consenting staff that are involve in nursing without any discrimination.

### Data analysis

Statistical analyzes were performed with EPI-INFO 3.5.4 and MS Excel. The significance level was set at 0.05 and all tests were two-sided. A simple descriptive analysis was performed to estimate the non-response rate; check the representativeness of the sample relative to the population of all HCWs of the YUTH; estimate proportions of gender, occupational categories and number of years of service. In addition, we determine the incidence rate and cumulated incidence of AEB. The proportions of victims were calculated as well as proportions of the different mechanisms involved (percutaneous injury or splash). Then we evaluated the risk of contamination for each type of AEB by determining the proportion of vaccinated or not against HVB amongst the victims, proportion of those who wore protective equipment at the moment of the accident (gloves, for example) as well as the proportion of victims who have benefited from a serological follow-up.

### Ethical approval

Written informed consent was sought prior to the inclusion in the study. This study was examined an approved by the Cameroon National Ethics Review Committee N° 2013/11/376/L/CNERSH/SP, including data collection tools and inform consent form.

## Results

### Demographic characteristics of respondents

Among the 325 HCWs of the YUTH, 233 were contacted and proposed to take part in the study, giving a recruitment rate of 71.69 %. One hundred and fifty HCWs accepted to participate in the study, thus giving a response rate of 64.38 %. Our sample was representative of all the HCWs of the YUTH, in terms of occupational categories and services as Table 1 shows. The only significant difference between population of the UTHY and the study population was higher proportion of HCWs in the study population who worked in the ICU.

**Table 1:** Representativeness of the study population compared to the population of the hospital

	Population of the UTHY		Study population		p value^a^
	Number	Proportion (%)	Number	Proportion (%)	
Services					
Intensive care units	44	13.54	32	21.33	0.0341
Surgery	34	10.46	23	15.33	0.1486
Emergency	23	7.08	12	8.00	0.8124
Pediatry	47	14.46	24	16.00	0.7744
Laboratory	54	16.61	19	12.67	0.3355
Gynecology	36	11.08	15	10.00	0.8163
Medicine	30	9.23	08	5.33	0.1977
Outpatient department	57	17.54	17	11.33	0.1480
Occupational category					
Physicians	62	19.08	29	19.33	0.9895
Nurses	98	30.15	59	39.33	0.0955
Nurses assistants	63	19.38	21	14.00	0.2245
Nurse-aids	75	23.08	29	19.33	0.4435
Laboratory technicians	27	8.31	12	8.00	0.9760
Total	325	100	150	100	

^a^As determined by the Chi square

The majority of the respondents were female (99; 66 %) and 71.33 % had at least 4 years of working experience. Table 2 presents general characteristics of the respondents.

**Table 2:** General characteristics of the respondents

Characteristics	Number	%
Sex		
Male	51	34.00
Female	99	66.00
Length of service (year)		
[0–3]	43	28.67
[4–6]	52	34.67
[7–10]	27	18.00
11 and more	28	18.67
Oldness in the service (year)^a^		
[0–3]	94	62.67
[4–6]	31	20.67
[7–10]	13	8.67
11 and more	11	7.33

^a^This refers to the number of years of experience in the service of the hospital in which the health worker works

### Knowledge on diseases resulting from AEB and vaccination against hepatitis B virus

One hundred and forty nine (99.3 %) and 146 (97.3 %) HCWs identified HIV and HBV as viruses that can be transmitted during and AEB respectively. Moreover, (20) 13.3 % and (57) 38 % of the respondents thought that influenza and tuberculosis can be transmitted following to and AEB. These last proportions are higher in nurse-aids compares to other job categories (X^2^ = 26, p < 0.0001) and (X^2^ = 57.42, p < 0.0001) respectively for influenza and tuberculosis.

In addition, 11.33 % (CI 6.25–16.40) of the respondents did not identified vaccination of caregivers as a measure to prevent transmission of blood-borne disease after and AEB. At the time the study was conducted, 55 HCWs (36.67 %) reported having received all the 3 doses of the vaccine against hepatitis B virus, 29 (19.33 %) have started but not yet finished the vaccination series and 66 (44 %) were not vaccinated. Physicians were more often immunized (48.3 %) in comparison to other job categories, nurses (35.6 %), nurses assistants (38.1 %) laboratory technicians (33.3 %) and nurse-aids (27.6 %), (X^2^ = 29.55; p < 0.0001).

### Frequency and nature of exposure

Fifty five healthcare workers (36.7 %) reported in total, 73 AEB in the previous 3 months prior to data collection. The most important exposure being splash (n = 44; 60.27 %), followed by needle stick (n = 21; 28.77 %), and cuts (n = 8; 10.9 %), as presented in Table 3.

**Table 3:** Frequency of types of AEB

Type of AEB	Number of cases	Proportion (%)	Mean number of AEB ± SD	Cumulated incidence (%)	Incidence rate (AES/100HCWs/year)
Needle stick	21	28.77	1.47 ± 0.928	14.00	18.67
Cut	8	10.96	1.5 ± 0.925	5.33	7.11
Splash	44	60.27	2.43 ± 1.731	29.33	39.11
Total	73	100	2.69 ± 2.044	48.67	64.89

Accidents distribution by the unit types where HCWs worked shows difference in location where the accident occurred, that is, accidents occurred more often in the surgical Department (20 %) than in other wards: intensive care units (18 %), pediatrics (16 %), emergency (11 %), gynecology (9 %). However, these differences were not statistically significant (p = 0.2310).

It may be noted that 75 % of victims of needle stick injury were by a hollow needle contaminated with blood. Also, 41 % of splashes occurred on injured skin. All reported cuts were superficial.

Accident distribution per job categories presented in Table 4 shows no statistically significant difference in the job category most affected by AEB, χ^2^ = 7.8689, p = 0.0965.

**Table 4:** Accidents distribution per job category

Job category	Number (%)			Mean number of AEB ± SD
	Accidents		Total	
	Yes	No		
Physicians	9 (16.4)	20 (21.1)	29 (19.3)	1.5 ± 0.849
Nurses	24 (43.6)	35 (36.8)	59 (39.3)	2.87 ± 1.825
Nurses assistants	8 (14.5)	13 (13.7)	21 (14)	2.5 ± 1.414
Nurse-aids	11 (20)	18 (18.9)	29 (19.3)	3.41 ± 3.204
Laboratory technicians	3 (5.5)	9 (9.5)	12 (8)	1.0 ± 0
Total	55 (36.7)	95 (63.3)	150 (100)	2.69 ± 2.044

### Use of protective equipment

Globally, 7.3 % of victims were not wearing gloves at the time of the exposure. More precisely, 14.3 % of victims of needle stick injury as well as 12.5 % of victims of cut were not wearing gloves. No eye shield or face shield was worn during execution of procedures at the time splash exposures occurred in 84.1 % of cases.

### Management of AEB

Cleaning the injury site with running water was the most frequently used first aid measure in over 80 % (n = 44) of HCWs injured. Other measures used for immediate management included cleaning with antiseptic solution (74.5 %). However, 1.8 % of HCWs did not take any cleaning action concerning the injury. Fifty point nine percent of victims had their baseline HIV testing done, 25.5 % received PEP for HIV and 21.8 % received follow-up care.

### Risk assessment during AEB

Up to 87.5 % of the HCWs who sustained needle stick injury with hollow contaminated needle wore gloves during the accident and 28 % of them were not vaccinated against HBV prior to the event. Of these, an initial serological examination was performed in 75 % of people, and only 25 % received serological follow-up care. All those who were not wearing gloves during the accident (12.5 %), were not vaccinated against HBV and for 50 % of them, serological follow-up was programmed.

Forty-four percent (44 %) of victims of splash on injured skin were not vaccinated against HBV; and among these unvaccinated victims, 75 % performed an initial serological testing, and 25 % received serological follow-up and adequate prophylaxis. Thirty seven point five percent of victims of cuts were not vaccinated against HBV and 66 % of them received baseline testing. Among those that received baseline testing, only 33 % benefited follow-up care such as second testing after 3 months’ time as exposition to HIV is concerned or additional laboratory testing.

## Discussion

Fifty five (36.7 %) HCWs reported having been exposed to blood and body fluid at least once in the 3 months preceding the study. Splash was the most reported injury (in 60.3 % of cases), followed by needle stick (28.7 %) and cuts (10.9 %). Moreover, the majority of victims (20 %), belong to the surgical department and only 36.6 % of respondents were vaccinated against hepatitis B.

The proportion of victims of AEB (36.7 %) observed in this study is significantly lower than that (60.7 %) of a study published in 2010 and conducted in Cameroon in a hospital of a similar technical ward [11]; meaning there would be an improving trend. But the more likely explanation would be the methodological difference, notably the duration of observed period which is lower in our case. However, this proportion remains high compared to the 25 % found in 2013 in Kenya [20]. Reason for this difference could be the knowledge gap of HCWs identified in our study when considering diseases that can be transmitted following and AEB and identification of vaccination against hepatitis B virus as a protective measure. Indeed, Muhonja identify staff training on AEB as a protective factor for having and AEB. Increased incidence of AEB has been explained by the absence of displayed protocols and inadequate behavior of caregivers [21]. Thus continuous staff training, supervision and the availability of displayed protocols appear as interventions to be implemented to minimize risks for caregivers.

Nurses (including nurses assistants and nurse aids) had accidents more often than physicians, what is contrary to the published papers in both developed and less developed countries, reporting usually that physicians are more prone to injuries involving exposure to blood [22, 23]. However, there are studies with results similar to ours, that is, which have shown that nurses had the highest rate of accidents in comparison to all other categories of health care providers [19]. It is a well-established fact that physicians report accidents to the responsible persons much more rarely than other HCWs [19]. Underreporting rates of 22 to 82 % have been noted [24–26]. In our study, we noticed that almost all of HCWs had not officially reported their accidents (none of the AEB registered), but simply started investigations without any official reporting of the accident to persons responsible for that. It is possible that the frequency of accidents was higher than the one reported in this study, but that they considered the accidents insignificant and hence did not report them, which resulted in those accidents being unrecorded in this research.

Hepatitis B vaccination coverage among HCWs was low at 36.6 % (fully vaccinated). According to the WHO estimates, vaccination coverage varies from 18 % in Africa to 77 % in Australia and New Zealand [27]. Doctors were more likely to be vaccinated among the HCWs. However, confirmation of protection by antibody estimation was not done. There are many potential reasons for low HBV vaccine coverage, the most common being unavailability of the vaccine at the health facility. While the vaccine is available at the market at a cost, HCW have relied on provision by their institutions. However, there is a moderately good awareness among HCWs (88.67 % of respondents identified vaccination as an important mean to limit contamination after and AEB). Other potential reasons supported by other studies include busy schedules, and low risk perception [20, 28].

This study has certain limitations, including the possibility of recall bias because information on exposure was sought for the preceding 3 months. Also, the study has not documented exposures among mortuary workers and cleaning staff that are also at risk. Nevertheless, the findings can be useful in designing interventions to safe guard the health of health workers.

## Conclusions and recommendations

Exposure to blood was very common with blood splash emerging as the most common route of exposure. Post-exposure management is poorly adhered to with gross underreporting of the exposures (none of the accidents reported). Efficient strategies to protect HCWs from occupational exposures to blood and body fluids should be identified and implemented. Facilities should establish surveillance system for registering, reporting and management of occupational injuries and exposures. In addition, education of workers on risks and institution of standard operating procedure are crucial to safeguard their health. All HCWs should be trained, sensitized and updated on issues related to infection prevention and occupational risk reduction. Hepatitis B vaccination is recommended for HCWs and institutions should provide mandatory immunization programmes for their HCWs.

## Abbreviations

YUTH: Yaounde University Teaching Hospital
HCWs: healthcare workers
AEB: accidental exposure to blood and body fluids
HBV: hepatitis B virus
OR: odds ratio
SD: standard deviation
HCV: hepatitis C virus
HIV: human immunodeficiency virus

## References

[1] International Labour Organization. An ILO code of practice on HIV/AIDS and world of work. Geneva: ILO; 2014. http://www.ilocarib.org. Last accessed 15th Jan 2014.

[2] J. Siegel, E. Rhinehart, M. Jackson, L. Chiarello, and the Healthcare Infection Control Practices Advisory Committee: Guideline for Isolation Precautions: Preventing Transmission of Infectious Agents in Healthcare Settings. 2007; Available from: http://www.cdc.gov/hicpac/pdf/isolation/Isolation2007.pdf 10.1016/j.ajic.2007.10.007 PMC7119119 18068815

[3] Sepkowitz KA (1996). Occupationally acquired infections in health care workers. Ann Intern Med.

[4] Calver J (1997). Occupational health services. Am J Infect Control.

[5] Azap A, Ergonul O, Memikoglu K, Yesxilkaya A, Altunsoy A, Bozkurt G (2005). Occupational exposure to blood and body fluids among health care workers in Ankara, Turkey. Am J Infect Control..

[6] Dobie DK, Worthington T, Faroqui M, Elliot TSJ (2002). Avoiding the point. Lancet.

[7] Berry A, Greene ES (1992). The risk of needlestick-transmitted diseases in the practice of anesthesiology. Anesthesiology.

[8] Pruss UA, Rapiti E, Hutin Y (2005). Estimation of global burden of disease attributable to contaminated sharps injuries among healthcare workers. Am J Ind Med.

[9] Pruss UA, Rapiti E, Hutin Y (2003). Sharps injuries; global burden of diseases from sharps injuries to healthcare workers.

[10] Tarantola A, Koumare A, Rachline A (2005). A descriptive, retrospective study of 567 accidental blood exposures in healthcare workers in three West African countries. J Hosp Infect.

[11] Mbanya D, Ateudjieu J, Tayou CT, Moudourou S, Lobe MM, Kaptue L (2010). Risk factors for transmission of HIV in a hospital environment of Yaoundé, Cameroon. Int J Environ Res Public Health.

[12] World Health Organization (2002). Reducing risks, promoting healthy life.

[13] OMS. Prévention des infections nosocomiales: Guide pratique. 2nd éd. Genève; 2008.

[14] Villafranca V (2006). La prévention des risques professionnels a l’hôpital: pour une politique de promotion de la santé au travail.

[15] Shapiro C (1995). Occupational risk of infection with hepatitis B and hepatitis C virus. Surg Clin North Am.

[16] David HT, David YM (1997). Living with needle stick injuries. J Can Dent Assoc.

[17] Salihović P, Puvačić S (2004). Occupational exposure and prevention of viral hepatitis B in health care personnel in the Canton of Sarajevo. Med Arh.

[18] Gupta A, Anand S, Sastry J, Krisagar A, Basavaraj A, Bhat SM, Gupte N, Bollinger RC, AL Kakrani (2008). High risk for occupational exposure to HIV and utilization of post-exposure prophylaxis in a teaching hospital in Pune, India. BMC Infect Dis.

[19] Marković LJ (2013). Occupational exposures to blood and body fluids among health care workers at university hospitals. Srp Arh Celok Lek.

[20] Muhonja E (2013). Prevalence of factors associated with percutaneous injuries and splash exposures among health-care workers in a provincial hospital Kenya 2010. Pan Afr Med J.

[21] Nouetchognou JS (2014). Evaluation de la prévention des infections nosocomiales et des accidents avec exposition au sang au CHU de Yaoundé.

[22] Wicker S, Jung J, Allwinn R, Gottschalk R, Rabenau HF (2008). Prevalence and prevention of needlestick injuries among health care workers in a German university hospital. Int Arch Occup Environ Health.

[23] Foster TM, Lee MG, McGaw CD, Frankson MA (2010). Prevalence of needlestick injuries and other high risk exposures among healthcare workers in Jamaica. West Indian Med J.

[24] Tarantola A, Abiteboul D, Rachline A (2006). Infection risks following accidental exposure to blood or body fluids in health care workers: a review of pathogens transmitted in published cases. Am J Infect Control.

[25] Schmid K, Schwager C, Drexler H (2007). Needlestick injuries and other occupational exposures to body fluids amongst employees and medical students of a German university: incidence and follow-up. J Hosp Infect.

[26] Colombo C, Ledergerber F, Zysset F, Francioli P, Cavassini M, Blanchet CL (2010). Exposition au risque infectieux VIH, VHB et VHC chez le personnel des établissements de soins en Suisse de 2001 à fin juin 2008. BAG Bull.

[27] Hutin Y, Hauri A, Chiarello L, Cattlin M, Stilwell B (2003). Injection safety best practices development group—best infection control practices for intradermal, subcutaneous and intramuscular needle injections. Bull World Health Organ.

[28] Abdul RM, Munit AS, Salahuddin A (2007). Hepatitis B vaccination status and knowledge, attitude, practices of health care workers regarding hepatitis B and C in a tertiary care setting of Karachi. Pak Med Net.

